# Papillary Thyroid Carcinoma Arising Within a Mature Cystic Ovarian Teratoma With Synchronous Primary Thyroid Malignancy: A Case Report

**DOI:** 10.7759/cureus.114864

**Published:** 2026-08-20

**Authors:** Luis Munoz-Andrade, Cristian Guaman, Maria-Jose Vega, Carlos Alarcon, Gema Plaza

**Affiliations:** 1 Surgery, Centro Médico Muñoz y Andrade, Quito, ECU; 2 Surgical Oncology, Sociedad de Lucha Contra el Cáncer del Ecuador, Guayaquil, ECU; 3 Gynecologic Oncology, Sociedad de Lucha Contra el Cáncer del Ecuador, Guayaquil, ECU; 4 Surgery, Sociedad de Lucha Contra el Cáncer del Ecuador, Guayaquil, ECU

**Keywords:** encapsulated follicular variant, frozen-section limitations, mature cystic teratoma, ovarian teratoma malignancy, papillary thyroid carcinoma, radioactive iodine ablation, synchronous thyroid malignancy

## Abstract

Malignant transformation of mature cystic teratomas (MCT) of the ovary is uncommon, with squamous cell carcinoma being the most frequent histotype. Papillary thyroid carcinoma (PTC) arising from ectopic thyroid tissue within an ovarian teratoma is an exceptionally rare entity, with only a limited number of cases documented in the international literature.

We report the case of a 34-year-old woman with an incidentally discovered left para-ovarian cystic mass detected during routine gynecological surveillance following a prior hysterectomy for cervical intraepithelial neoplasia. The patient underwent laparoscopic left salpingo-oophorectomy; intraoperative frozen-section biopsy was negative for malignancy. However, definitive histopathological examination identified a papillary thyroid microcarcinoma, follicular variant, arising within a MCT of the left adnexa, without ovarian surface involvement or lymph node metastasis (pT1). This finding prompted formal thyroid evaluation, which revealed a synchronous primary PTC of the thyroid gland, encapsulated follicular variant, measuring 2.3 × 2 cm (pT2), requiring staged bilateral thyroid surgery and planned adjuvant radioactive iodine ablation.

This case illustrates the diagnostic challenge of occult thyroid carcinoma within ovarian teratomas, the limitations of intraoperative frozen-section analysis, and the critical importance of a multidisciplinary approach integrating gynecological surgery, endocrinology, and nuclear medicine. The diagnosis of teratoma-derived PTC should prompt the consideration of a formal thyroid evaluation. Thyroid ultrasonography is recommended, whereas serum thyroglobulin should be interpreted only as an adjunctive marker within the overall clinical context and should not be used in isolation for clinical decision-making.

## Introduction

Mature cystic teratomas (MCT), also known as dermoid cysts, are the most common germ cell tumors of the ovary, accounting for approximately 10-20% of all ovarian neoplasms [1]. These tumors are typically benign and contain well-differentiated tissue elements derived from all three embryonic layers: ectoderm (skin, hair follicles, and sebaceous glands), mesoderm (adipose tissue, muscle, and cartilage), and endoderm (gastrointestinal, respiratory, and thyroid tissue). Thyroid tissue is present in approximately 12% of MCT [2]. When thyroid tissue constitutes more than 50% of the lesion, the entity is classified as struma ovarii, a monodermal variant of teratoma that represents a distinct diagnostic category [3].

Malignant transformation of MCT is uncommon, with an estimated incidence of 0.17-2%; when it occurs, squamous cell carcinoma accounts for approximately 80% of malignant transformations [1,4]. Less frequent malignancies include carcinoid tumors, sarcomas, adenocarcinomas, and thyroid carcinomas. Papillary thyroid carcinoma (PTC) arising from thyroid elements within an ovarian teratoma is exceptionally rare, with fewer than 20 cases reported in the international literature [5-8]. The diagnosis is almost always established incidentally on definitive histopathology, as neither preoperative imaging nor intraoperative frozen-section analysis is sufficiently sensitive to identify the characteristic nuclear features of PTC in teratomatous thyroid tissue [5,9,10].

The coexistence of a teratoma-derived PTC and a synchronous primary thyroid carcinoma introduces additional diagnostic and therapeutic complexity. This unusual presentation raises questions about the independent versus metastatic nature of each lesion, the appropriate extent of surgical management, and the role of adjuvant radioactive iodine. We herein report the case of a papillary thyroid microcarcinoma, follicular variant, incidentally diagnosed within a mature cystic ovarian teratoma in a 34-year-old woman, who was subsequently found to harbor a synchronous primary PTC of the thyroid gland, encapsulated follicular variant, requiring a staged multidisciplinary approach.

## Case presentation

A 34-year-old woman with no significant past medical history presented for routine gynecological follow-up after a prior total hysterectomy performed for cervical intraepithelial neoplasia with squamous metaplasia of the endocervix. Consecutive post-surgical cytological controls were negative for intraepithelial lesion.

**Figure 1:**
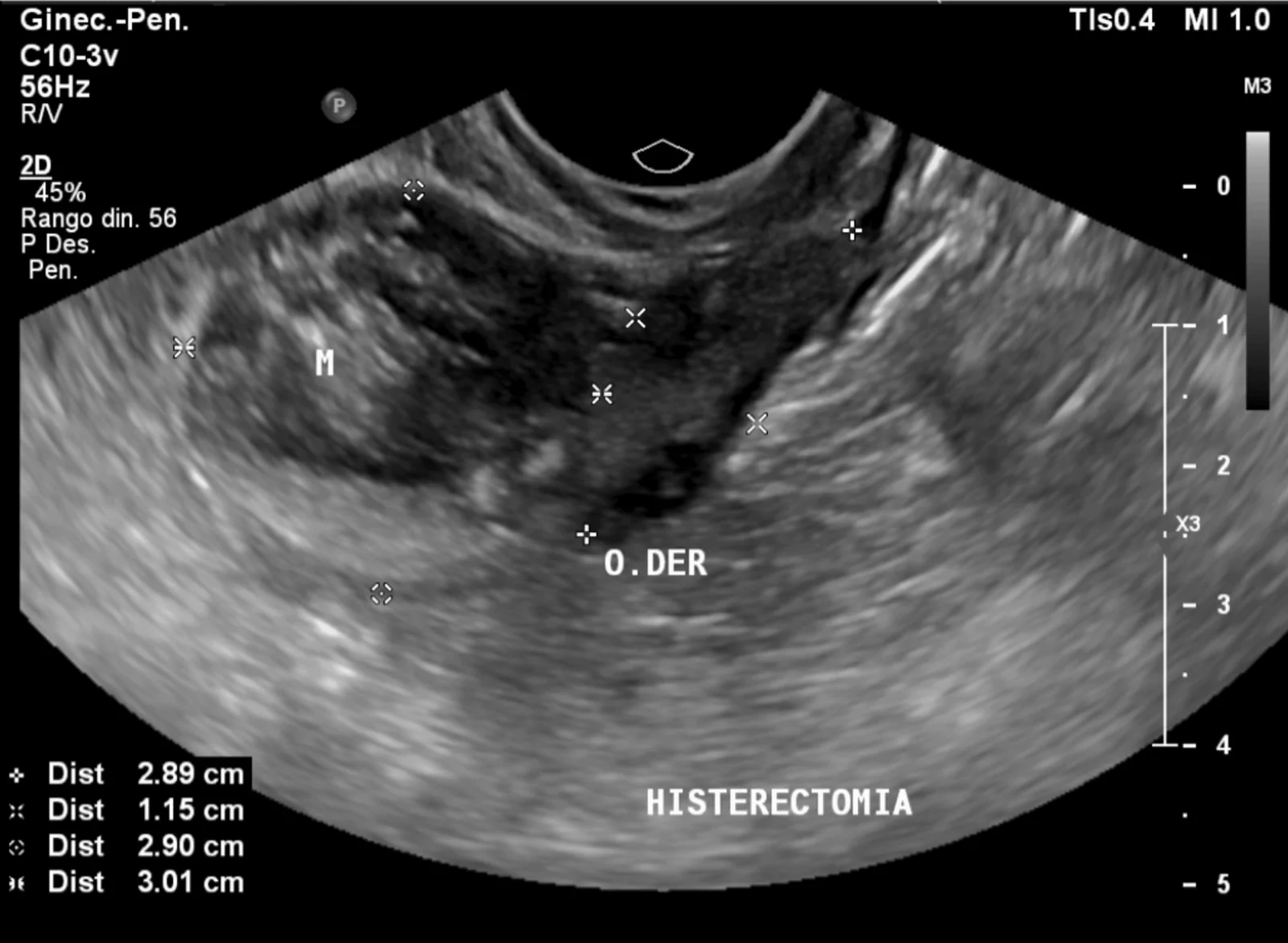
Initial endovaginal ultrasound showing a para-ovarian nodular image adjacent to the right ovary Endovaginal ultrasound demonstrating the right ovary (O.DER) with an adjacent heterogeneous, rounded para-ovarian nodular image with defined margins, measuring 2.89 × 2.90 × 3.01 cm, consistent with a dermoid cyst. The patient had previously undergone hysterectomy (noted on image).

One year after hysterectomy, routine endovaginal ultrasound revealed a heterogeneous tumoral lesion adjacent to the right ovary with partially defined margins measuring 2.9 × 2.18 cm (Figure 1).

**Figure 2:**
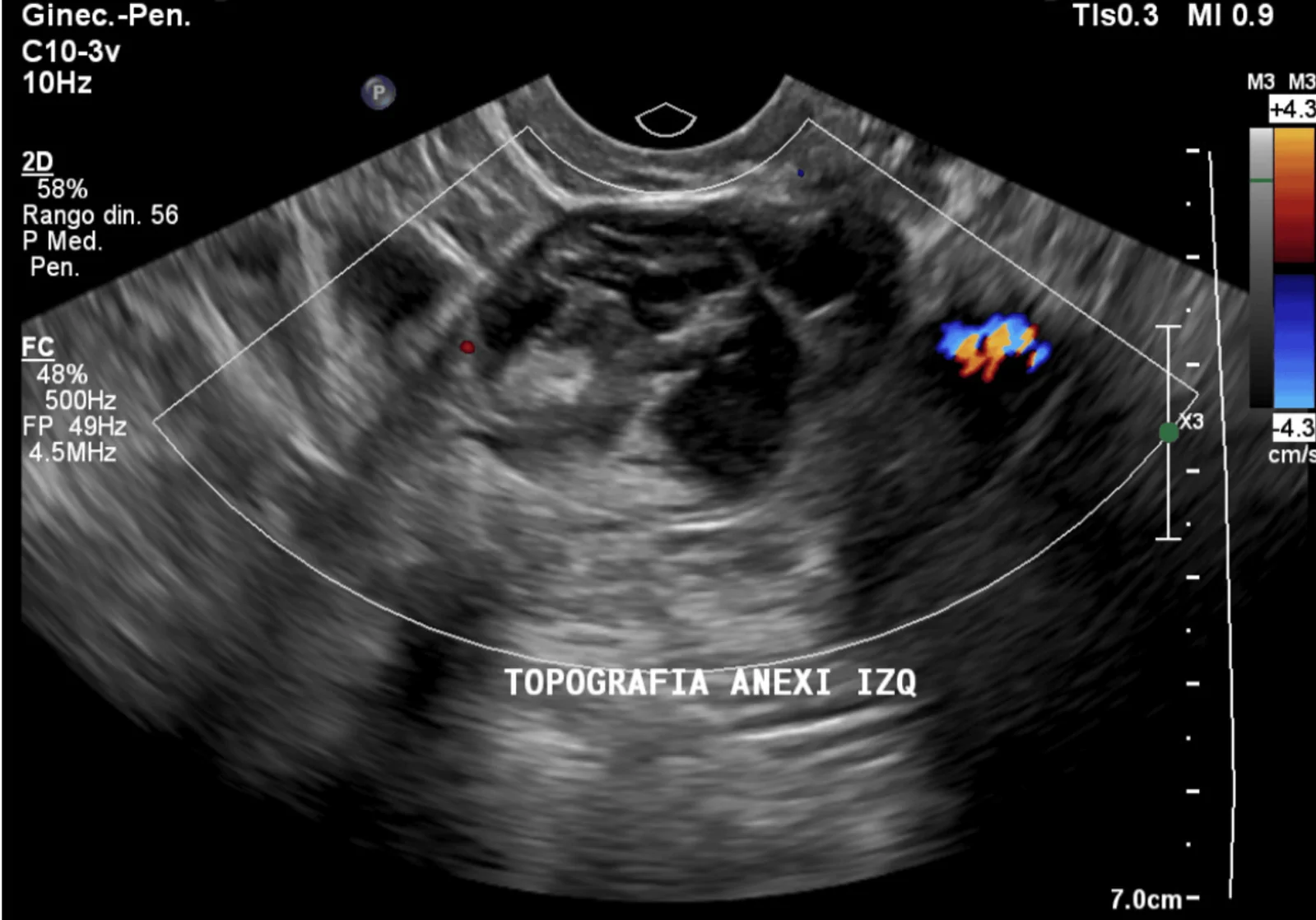
Follow-up endovaginal ultrasound demonstrating the interval growth of the left adnexal cystic mass Follow-up endovaginal ultrasound of the left adnexal region (TOPOGRAFIA ANEXI IZQ) showing a complex cystic mass with internal echoes and Doppler flow signal, with interval growth compared to prior imaging, prompting surgical intervention. The lesion measured 3.3 × 2.8 × 3.1 cm on this examination.

Pelvic magnetic resonance imaging (MRI) reported a left adnexal image of 2 × 4 cm, interpreted as a probable hemorrhagic cyst. Tumor markers cancer antigen 125 (CA-125) and human epididymis protein 4 (HE4) were within normal limits. In the absence of imaging criteria for malignancy, the patient was maintained under clinical and radiological surveillance. Annual ultrasound follow-up demonstrated the progression of the left para-ovarian lesion to a complex cystic mass of 3.3 × 2.8 × 3.1 cm, prompting surgical intervention (Figure 2).

**Figure 3:**
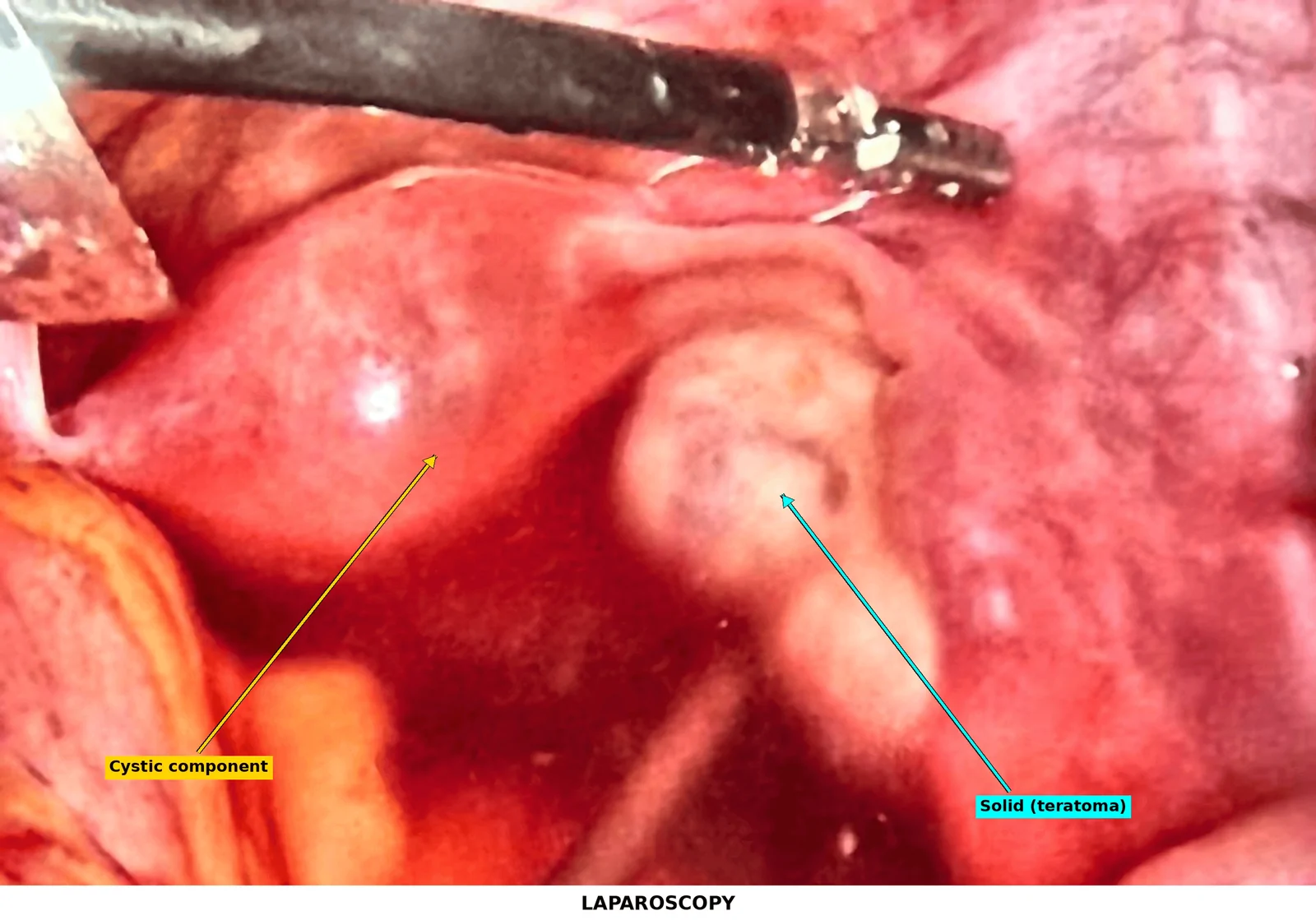
Intraoperative laparoscopic image of the left adnexal mass Laparoscopic view showing the left para-ovarian solid-cystic mass. Yellow arrow indicates the cystic component; cyan arrow indicates the solid component, later identified histopathologically as a mature cystic teratoma containing a papillary thyroid microcarcinoma.

The patient underwent laparoscopic left salpingo-oophorectomy (Figure 3).

Intraoperative findings revealed a solid-cystic left para-ovarian mass of 3 cm, submitted for frozen-section biopsy, which reported negative for malignancy and compatible with teratoma. The postoperative course was uneventful, and the patient was discharged at 24 hours. However, definitive histopathological examination identified a papillary thyroid microcarcinoma, follicular variant, arising within a MCT of the left adnexa. The lesion measured less than 1 cm and showed sheets of polygonal cells with eosinophilic to vacuolated cytoplasm, minimal atypia, absence of Reinke crystals, and nuclear features consistent with PTC. There was no involvement of the ovarian surface or the fallopian tube. Regional lymph nodes (0/7) and peritoneal washings were negative for malignancy, yielding a pathological staging of pT1.

**Figure 4:**
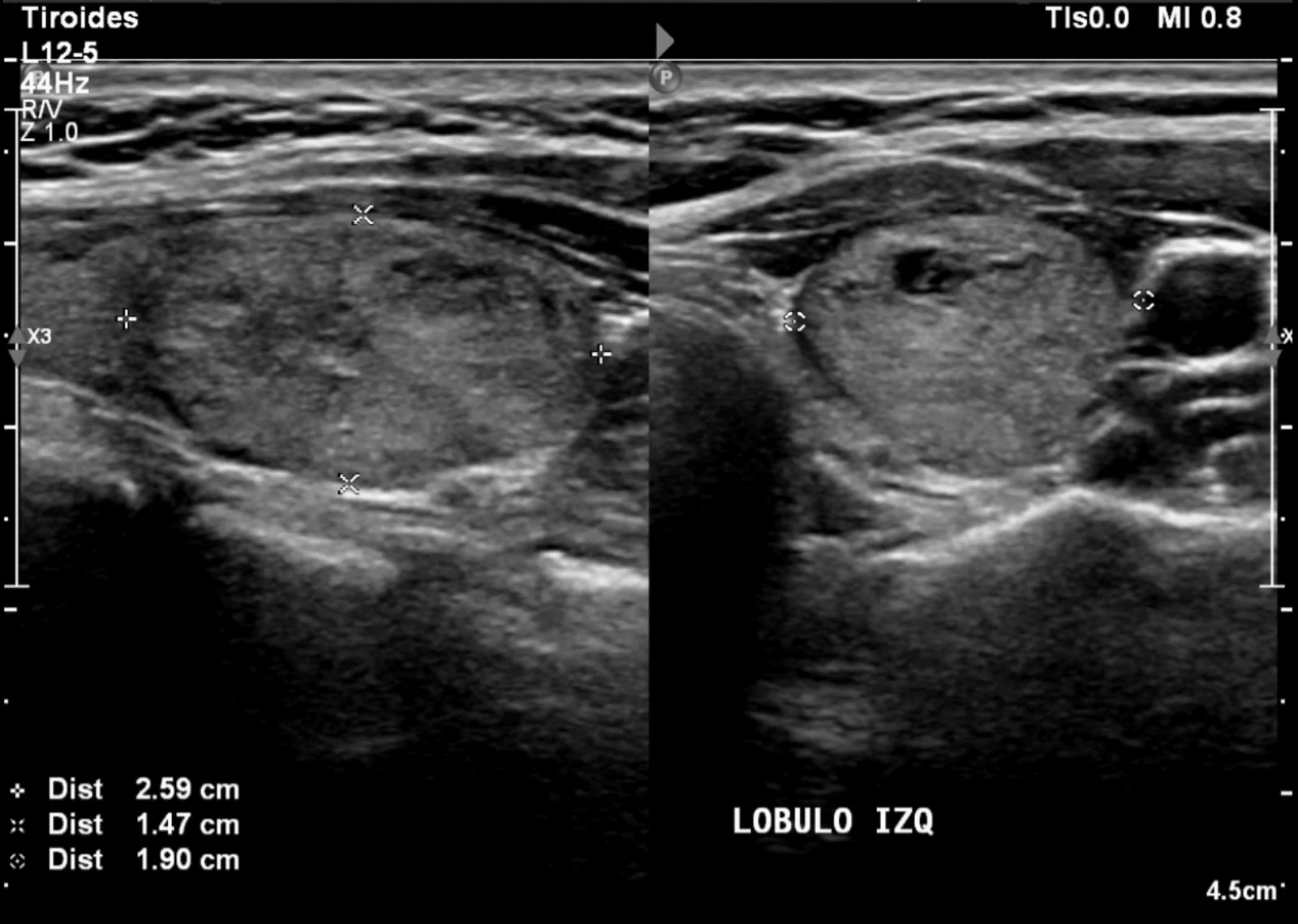
Thyroid ultrasound showing a TI-RADS 4A nodule in the left thyroid lobe Thyroid ultrasound (LOBULO IZQ) demonstrating a heterogeneous solid nodule with a hypoechoic halo, mixed solid-cystic components, and circumscribed margins, measuring 2.59 × 1.47 × 1.90 cm, located at the junction of the middle and lower thirds of the left thyroid lobe. The nodule was classified as TI-RADS 4A. Fine-needle aspiration biopsy returned Bethesda II; however, serum thyroglobulin was elevated at 30 ng/mL, prompting surgical resection. Definitive pathology identified a papillary thyroid carcinoma, encapsulated follicular variant (pT2). TI-RADS: Thyroid Imaging Reporting and Data System

Following this finding, a comprehensive thyroid evaluation was initiated. Thyroid ultrasound demonstrated a heterogeneous solid nodule with a hypoechoic halo, mixed solid-cystic components, circumscribed margins, and central and peripheral vascularity, measuring 2.5 × 1.4 × 1.9 cm at the junction of the middle and lower thirds of the left lobe, classified as Thyroid Imaging Reporting and Data System (TI-RADS) 4A (Figure 4).

**Figure 5:**
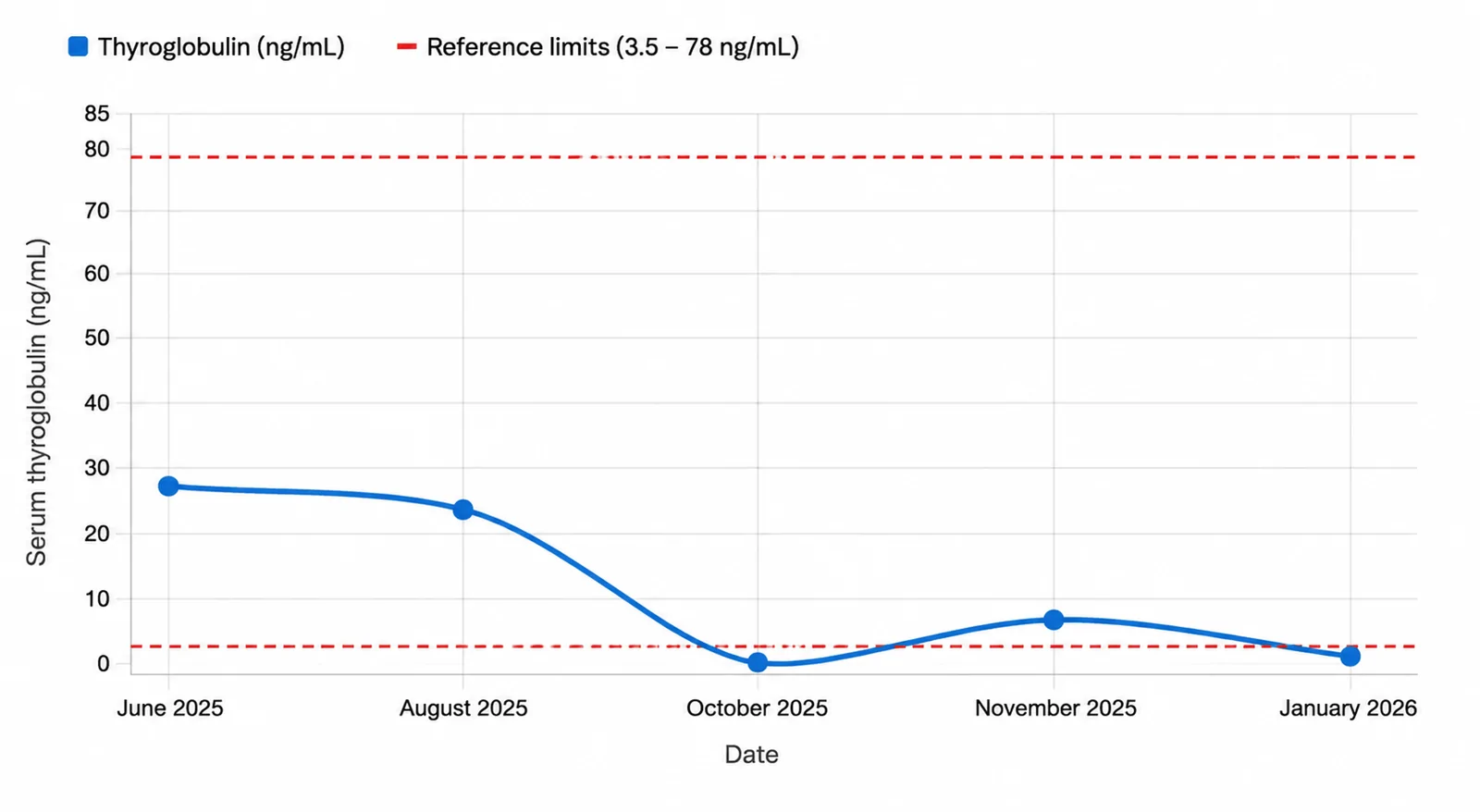
Serial serum thyroglobulin levels throughout the surgical treatment course Line graph depicting serial serum thyroglobulin measurements from June 2025 to January 2026. The initial pre-surgical value was 28 ng/mL, followed by a decline to 7.53 ng/mL after left thyroid loboisthmusectomy (November 2025) and near-undetectable levels of approximately 1 ng/mL following completion thyroidectomy (January 2026). The pink line represents the upper limit of normal (78 ng/mL); the lower reference limit is 3.5 ng/mL. The progressive decline in thyroglobulin confirms the adequate surgical resection of thyroid tissue in preparation for radioiodine ablation.

Fine-needle aspiration biopsy (FNAB) of the thyroid nodule returned a Bethesda II classification (benign cytology compatible with nodular follicular disease). Despite the benign cytology (Bethesda II), serum thyroglobulin measured 30 ng/mL. This finding was interpreted as an adjunctive element within the overall clinical context and was not used in isolation to determine surgical management (Figure 5).

Given the elevated thyroglobulin in the clinical context of a synchronous ovarian PTC, the multidisciplinary team decided to proceed with surgical resection. The patient underwent left thyroid loboisthmusectomy; intraoperative frozen-section biopsy was again negative for malignancy. Definitive pathological examination identified a unifocal PTC, encapsulated follicular variant, measuring 2.3 × 2 cm, without lymphovascular invasion. The remnant right lobe and isthmus were free of malignancy. The lesion was staged as pT2 according to the eighth edition of the American Joint Committee on Cancer (AJCC) TNM classification.

**Figure 6:**
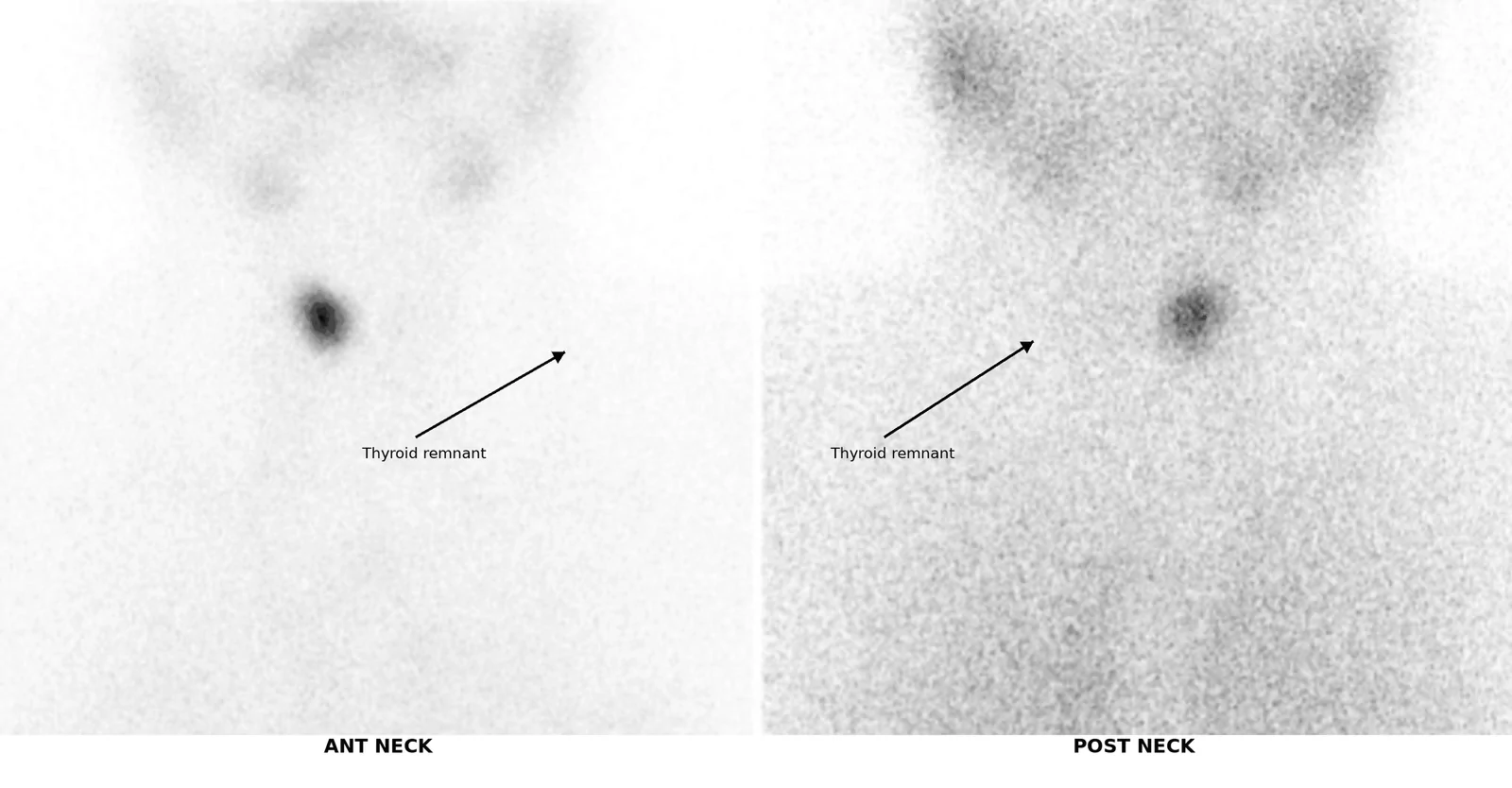
Thyroid scintigraphy demonstrating focal remnant thyroid tissue in the right thyroid lobe Anterior (ANT NECK) and posterior (POST NECK) thyroid scintigraphy images obtained after the administration of 5 mCi of 99mTc-pertechnetate, following left thyroid loboisthmusectomy. A focal area of increased uptake is identified in the topography corresponding to the superior pole of the right thyroid lobe, consistent with residual thyroid tissue of limited extension. This finding confirmed incomplete thyroidectomy and prompted completion right loboisthmusectomy to optimize conditions for subsequent radioiodine (^131^I) ablation.

The case was evaluated at a multidisciplinary oncological board with the participation of endocrinology and nuclear medicine. The patient was classified as low risk for recurrence, and adjuvant ablative treatment with 30 mCi of iodine-131 (¹³¹I) was indicated. However, thyroid-stimulating hormone (TSH) stimulation during the pre-ablation period did not reach adequate elevation. Thyroid scintigraphy was compatible with focal thyroid remnants of limited extension in the superior pole of the right thyroid lobe (Figure 6).

To optimize conditions for radioiodine ablation, the patient underwent completion right thyroid loboisthmusectomy. Anatomopathological study of the right lobe revealed a follicular adenoma and chronic thyroiditis, with no residual malignancy. The postoperative course was uneventful. Follow-up with imaging and serum thyroglobulin was scheduled according to institutional oncological protocol.

## Discussion

PTC arising within a mature cystic ovarian teratoma is one of the rarest forms of malignant transformation of ovarian germ cell tumors. A 2023 literature review cataloged fewer than 20 cases worldwide, with a mean age of approximately 44 years, although cases in younger women have been described [5]. Our patient, diagnosed at age 34, falls within this younger subgroup and is consistent with prior reports describing this entity in the reproductive-age population [8,9,11]. In the majority of published cases, including ours, the diagnosis was entirely incidental: established on definitive histopathology after surgery performed for an ovarian mass identified on imaging, with no preoperative suspicion of malignancy [5,6].

A central diagnostic challenge in this case was the false-negative result of the intraoperative frozen-section biopsy. Rapid intraoperative processing has well-documented limitations for identifying PTC in teratomatous thyroid tissue, as the characteristic nuclear features of PTC, nuclear clearing, grooves, pseudoinclusions, and psammoma bodies, may be subtle and require the careful examination of paraffin-embedded sections [5,9]. This is especially true for the encapsulated follicular variant, which lacks papillary architecture and relies on nuclear morphology for its diagnosis, a feature particularly susceptible to interpretive difficulties in frozen tissue [12,13]. Exhaustive microscopic sampling of thyroid elements present in teratomas, on definitive sections and complemented with immunohistochemistry for thyroid transcription factor-1 (TTF-1) and thyroglobulin when appropriate, is therefore essential to exclude occult malignant transformation in this tissue component [4,5].

The histological subtype identified in the ovarian lesion, PTC microcarcinoma, follicular variant (maximum dimension ≤1 cm), is generally associated with an excellent prognosis. The absence of lymphovascular invasion, ovarian surface involvement, and regional or peritoneal disease at the time of salpingo-oophorectomy corresponds to a confined pT1 stage, consistent with the favorable outcomes reported for PTC arising in ovarian teratomas [8,9,11]. In isolated cases of teratoma-derived PTC without extra-ovarian extension, conservative management with surveillance has been proposed in selected patients; however, when concurrent thyroid pathology is identified, management decisions must be individualized within a multidisciplinary framework [5,11].

The most distinctive finding of this case is the synchronous discovery of a primary PTC (encapsulated follicular variant, 2.3 cm, pT2) in the thyroid gland. The coexistence of a teratoma-derived PTC and a synchronous primary thyroid PTC is exceptional, with only isolated cases reported in the literature [6,7,12]. A recent Ecuadorian case report described a metachronic sequence, PTC in struma ovarii followed three years later by a thyroid PTC, highlighting the diagnostic and therapeutic dilemma of distinguishing primary from metastatic disease [13]. In our patient, the synchronous presentation, the confined ovarian staging (pT1, negative lymph nodes, negative peritoneal washings), and the independently encapsulated morphology of the thyroid carcinoma collectively support the existence of two independent primary tumors rather than ovarian metastasis from a thyroid primary [5,9]. This is consistent with the known potential for independent multifocal oncogenesis in follicular thyroid epithelium, including ectopic thyroid tissue [3,11].

Regarding thyroid management, the decision to proceed with left thyroid lobectomy despite a Bethesda II FNAB result was based primarily on the suspicious ultrasonographic features and the overall clinical context of a patient with synchronous teratoma-derived PTC. Serum thyroglobulin was considered only as a complementary finding and not as an independent indication for surgery. This underscores the clinical utility of thyroglobulin as a complementary marker and the importance of clinical context in surgical decision-making, beyond isolated cytological classification [14]. The intraoperative frozen-section biopsy of the thyroid was again falsely negative, a recognized limitation of this technique in the encapsulated follicular variant of PTC, for which deferred sections and, frequently, immunohistochemistry or molecular analysis are necessary for definitive classification [13]. Completion right thyroid loboisthmusectomy was subsequently performed to achieve total thyroidectomy in preparation for radioiodine ablation, with the right lobe demonstrating only follicular adenoma and chronic thyroiditis without residual malignancy. The planned adjuvant ablation with ¹³¹I (30 mCi) was individualized based on the presence of a pT2 primary thyroid PTC and the need for remnant thyroid tissue ablation to enable long-term thyroglobulin-based surveillance [14].

## Conclusions

This case illustrates the rare but clinically significant occurrence of PTC arising within a mature cystic ovarian teratoma, incidentally diagnosed on definitive histopathology following laparoscopic salpingo-oophorectomy, with the simultaneous finding of a synchronous primary PTC of the thyroid gland. Key takeaways include the following: First, intraoperative frozen-section biopsy is unreliable for detecting PTC in teratomatous thyroid tissue and cannot substitute for exhaustive paraffin-section analysis with immunohistochemical confirmation. Second, patients diagnosed with teratoma-derived PTC should undergo individualized thyroid evaluation. Thyroid ultrasonography appears to be an appropriate first-line investigation, whereas serum thyroglobulin should be interpreted cautiously as an adjunctive biomarker within the overall clinical context rather than as a standalone diagnostic tool. Third, when a synchronous thyroid malignancy is identified, management should follow established principles for differentiated thyroid carcinoma within a multidisciplinary framework. Lastly, clinical context, including thyroglobulin levels, should complement cytological classification in surgical decision-making. Given the rarity of this entity, additional well-documented cases with long-term follow-up and, when available, molecular characterization are needed before broad diagnostic or management recommendations can be established.

## References

[1] Hackethal A, Brueggmann D, Bohlmann MK, Franke FE, Tinneberg HR, Münstedt K (2008). Squamous-cell carcinoma in mature cystic teratoma of the ovary: systematic review and analysis of published data. Lancet Oncol.

[2] Devaney K, Snyder R, Norris HJ, Tavassoli FA (1993). Proliferative and histologically malignant struma ovarii: a clinicopathologic study of 54 cases. Int J Gynecol Pathol.

[3] Hinshaw HD, Smith AL, Desouki MM, Olawaiye AB (2012). Malignant transformation of a mature cystic ovarian teratoma into thyroid carcinoma, mucinous adenocarcinoma, and strumal carcinoid: a case report and literature review. Case Rep Obstet Gynecol.

[4] Qaradakhy AJ, Ali RM, Ali RM (2025). Papillary thyroid carcinoma within struma ovarii: a case report and literature review. J Surg Case Rep.

[5] Hmidi A, Sahraoui G, Charfi L, Hassine A, Aloui H, Mrad K (2023). Unexpected finding: papillary thyroid carcinoma arising in a mature ovarian teratoma - a case report and literature review. Int J Surg Case Rep.

[6] Al-Janabi MH, Khaddour T, Salloum R (2023). Papillary thyroid carcinoma arising from a mature ovarian teratoma coexisting with stromal luteoma: the first case report in the literature. J Surg Case Rep.

[7] Ferrer MS, Costa G (2017). Tejido tiroideo con carcinoma papilar en teratoma quístico maduro de ovario. Rev Venez Oncol.

[8] Ouviña Millán O, González Gil PM (2011). Malignant transformation of thyroid tissue in a mature ovarian cystic teratoma. Prog Obstet Ginecol.

[9] Dorado-Roncancio EF, Carrillo-Garibaldi OJ (2017). Malignant transformation of an ovary mature cystic teratoma: case report. Ginecol Obstet Mex.

[10] Vorasart P, Aroonroch R, Rermluk N, Vallibhakara O, Sriphrapradang C (2025). Papillary thyroid carcinoma arising within a mature ovarian cystic teratoma: a case report. Case Rep Endocrinol.

[11] Semanate F, Montoya S, Andrade E, Palacios C, Fernandez Trokhimtchouk T (2025). Papillary thyroid carcinoma arising in struma ovarii with subsequent thyroid microcarcinoma: a case report and review of the literature. Cureus.

[12] Haugen BR, Alexander EK, Bible KC (2016). 2015 American Thyroid Association management guidelines for adult patients with thyroid nodules and differentiated thyroid cancer: the American Thyroid Association guidelines task force on thyroid nodules and differentiated thyroid cancer. Thyroid.

[13] Stanković ZB, Djukić MK, Sedlecki K, Djuricić S, Lukac BJ, Mazibrada I (2006). Rapidly growing bilateral ovarian cystadenoma in a 6-year-old girl: case report and literature review. J Pediatr Adolesc Gynecol.

[14] Obwegeser R, Deutinger J, Bernascheck G (1993). The risk of malignancy with an apparently simple adnexal cyst on ultrasound. Arch Gynecol Obstet.

